# Examining the Evidence for Regulated and Programmed Cell Death in Cyanobacteria. How Significant Are Different Forms of Cell Death in Cyanobacteria Population Dynamics?

**DOI:** 10.3389/fmicb.2021.633954

**Published:** 2021-03-22

**Authors:** Daniel J. Franklin

**Affiliations:** Centre for Ecology, Environment and Sustainability, Department of Life and Environmental Sciences, Faculty of Science and Technology, Bournemouth University, Poole, United Kingdom

**Keywords:** growth, loss, biotic interactions, mortality, population dynamics

## Abstract

Cyanobacteria are ancient and versatile members of almost all aquatic food webs. In freshwater ecosystems some cyanobacteria form “bloom” populations containing potent toxins and such blooms are therefore a key focus of study. Bloom populations can be ephemeral, with rapid population declines possible, though the factors causing such declines are generally poorly understood. Cell death could be a significant factor linked to population decline. Broadly, three forms of cell death are currently recognized – accidental, regulated and programmed – and efforts are underway to identify these and standardize the use of cell death terminology, guided by work on better-studied cells. For cyanobacteria, the study of such differing forms of cell death has received little attention, and classifying cell death across the group, and within complex natural populations, is therefore hard and experimentally difficult. The population dynamics of photosynthetic microbes have, in the past, been principally explained through reference to abiotic (“bottom-up”) factors. However, it has become clearer that in general, only a partial linkage exists between abiotic conditions and cyanobacteria population fluctuations in many situations. Instead, a range of biotic interactions both within and between cyanobacteria, and their competitors, pathogens and consumers, can be seen as the major drivers of the observed population fluctuations. Whilst some evolutionary processes may theoretically account for the existence of an intrinsic form of cell death in cyanobacteria, a range of biotic interactions are also likely to frequently cause the ecological incidence of cell death. New theoretical models and single-cell techniques are being developed to illuminate this area. The importance of such work is underlined by both (a) predictions of increasing cyanobacteria dominance due to anthropogenic factors and (b) the realization that influential ecosystem modeling work includes mortality terms with scant foundation, even though such terms can have a very large impact on model predictions. These ideas are explored and a prioritization of research needs is proposed.

## Growth and Loss in Aquatic Photosynthetic Microbes: Important Biotic Interactions Overlie the Abiotic Fundamentals

Explaining patterns in aquatic primary production has always been an important scientific goal. Now, understanding differences in aquatic production across space and time is more important than ever given concern over the human alteration of the global carbon cycle. Explaining the major trends in production through reference mainly to abiotic factors (i.e., temperature, light and nutrients) has been a core endeavor in marine systems (Sverdrup, 1953; Boyd et al., 2014) though in recent years, as data accumulates and observation technologies improve, the importance of biotic interactions such as the close coupling between production and grazing (Behrenfeld, 2010; Banse, 2013), and the role of viral lysis (Talmy et al., 2019) has been increasingly recognized. Such work builds on the revolution in understanding afforded by the microbial loop paradigm (Azam et al., 1983) and the discovery of the sophisticated and complex lives of the small, diverse marine microbes (the picoeukaryotes and picocyanobacteria) which were invisible until the 1980’s (Chisholm et al., 1988). Recently, long-term observations (Hunter-Cevera et al., 2020) indicate that the majority of cell losses in populations of the ubiquitous picocyanobacteria *Synechococcus* are due to ecological interactions (grazing and viral lysis) and in the open ocean, the persistence, as well as the proliferation, of *Prochlorococcus* is intimately linked to complex biotic interactions with heterotrophic bacteria (Morris et al., 2011; Roth-Rosenberg et al., 2020). Our knowledge of death rates in *Prochlorococcus*, which can be shown by inference to be sizable, be it by viral infection, predation, or “spontaneous cell death” is in its infancy (Biller et al., 2014). Some marine cyanobacteria, such as *Trichodesmium* (“sea-sawdust”), are noted for their ability to form blooms in the tropics and subtropics and *Trichodesmium* population dynamics are thought to involve a form of programmed cell death (PCD) (Berman-Frank et al., 2004). More recently, the Nomenclature Committee on Cell Death has issued recommendations on the use of the terms accidental, regulated, and PCD and their recognized subvariants (Kroemer et al., 2009; and see Aguilera et al. this volume).

## Population Growth and Loss in Freshwater Cyanobacteria

**FIGURE 1:**
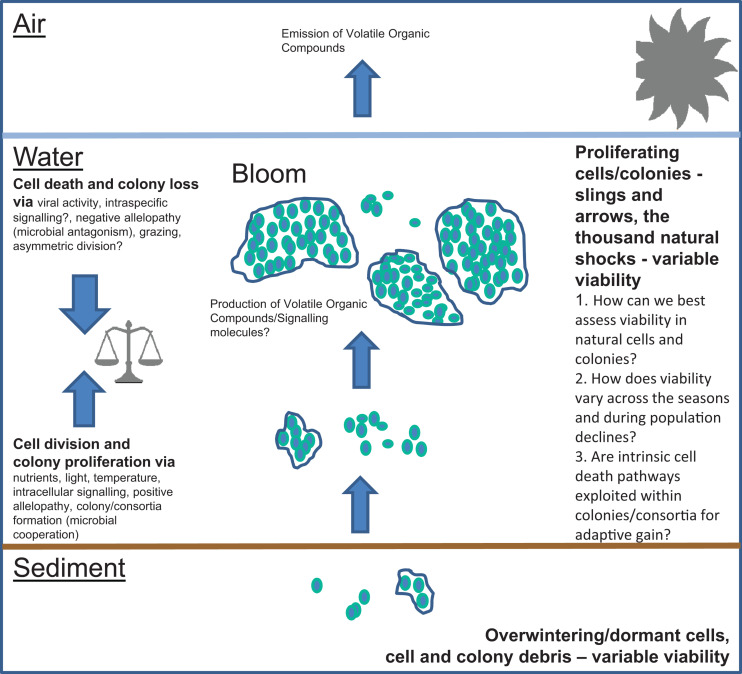
Factors important in the population dynamics of freshwater cyanobacteria. Important areas of uncertainty are (1) How can we best assess viability in natural cells and colonies?, (2) How does viability vary across the seasons and during population declines? And (3) Are intrinsic cell death pathways exploited within colonies/consortia for adaptive gain?

The diversity, ecophysiology and population dynamics of freshwater cyanobacteria has been studied for longer than the marine cyanobacteria due to their accessibility and because of the serious water management problems they can create. In addition, many freshwater cyanobacteria are generally larger and colony-forming compared with the marine forms. For the <30 or so freshwater cyanobacteria that can cause nuisance blooms (Whitton and Potts, 2013) much effort has been focused on the development of ecological models which predict blooms for better water resource management (e.g., Carvalho et al., 2011; Oliver et al., 2013). Abiotic factors are considered as major factors in such models, whereas complex biotic interactions, and, potentially, intrinsic mortality, are both much harder to understand and constrain for modeling purposes (Oliver et al., 2013). Ecological modelers typically include terms for cyanobacteria mortality, usually set to rather small values, and which receive little attention. Despite this, mortality, as a fundamental in population dynamics, can drastically determine the ecosystem responses which are the focus of such modeling efforts (Munkes et al., unpublished). Modeling therefore requires more information on the factors that lead to cell death and how these factors commonly influence microbial population dynamics. Figure 1 presents a summary of the factors involved in cyanobacteria population dyanamics and the unknowns which merit further study.

## Case Study: *Microcystis* Mortality During Life-History – Intrinsic Causes Vs. External Factors. Can We Recognize the Differences?

### Intrinsic Causes

*Microcystis* is probably the most well studied of the freshwater cyanobacteria. Detailed long-term studies have documented the complexity of *Microcystis* life-history (see Reynolds, 2006) with reference to physiological variability (in terms of ultrastructure and pigmentation) over seasonal transitions. For example, the summer-autumn transition in the temperate zone can involve a population-level switch from active proliferation in the water column to preparation for over-wintering, or “resting,” in the sediment. To some extent, such transitions may be seen as a coordinated aspect of *Microcystis* life-history and it has been suggested that the summer-autumn transition could involve the mortality of some cells such that the potential survival (persistence) of other cells is favored (Sirenko, quoted by Reynolds et al., 1981; see Reynolds, 2006). Whilst the persistence, via metabolic down-regulation (quiescence), of benthic over-wintering *Microcystis* cells is a well-documented strategy (e.g., LaTour et al., 2004a,b; Kitchens et al., 2018) it is difficult to see how preparation at the population level for such persistence could be causally linked to the death of conspecific cells unless through the operation of an adaptive differentiation process which, as a by-product, also produces dead cells. Within cyanobacteria there are several precedents for differentiation within colonies producing dead cells (see references in Franklin, 2014). Linked to this idea, it may be that during the transition period, sensed by *Microcystis* cells via environmental cues, some cells are triggered to bequeath an unequal allocation of resources during division (i.e., in a form of asymmetric division; reviewed in Franklin, 2014). This unequal allocation of resources may maximize the persistence of some of the cells whilst leading to the cell death of others as part of an adaptive response to changing conditions. Asymmetric division may be widespread amongst bacteria (Stewart et al., 2005), where it is also linked to the management of some forms of metabolic waste. Evolution has generated strategies to manage the accumulation of cellular damage, from for example, reactive oxygen species (ROS), within microbial populations as damage translates directly into fitness (Lindberg and Collins, 2020). Asymmetric division could help explain differential responses within microbial populations to environmental challenges, as well as the constant death rates that can be observed in steady-state continuous cultures (Lee and Rhee, 1999). Surveying the proportion of non-viable (dead) cells is a difficult and time-limited technical challenge though it is clearly the first step in the investigation of the extent to which the need to adapt to abiotic change at the population level may have resulted in adaptive, and truly intrinsic, forms of cell death. The technical difficulties of completing such surveys have made them rare and different methodologies have been used which assess different aspects of individual cell physiological state. In a temperate shallow water body very high proportions of *Microcystis* cells can be shown to be dead (SYTOX-green labeling) during the autumn transition (Kozik et al., 2019). The presence of variable amounts of dead cells (Evan’s blue staining) in *Microcystis* colonies was also noted in a similar water body during the summer-autumn transition, and the cause of death was thought to be adverse environmental conditions. In this case cell death was also linked to an intrinsic cell death pathway because DNA fragmentation (assessed via TUNEL labeling) was detected (Sigee et al., 2007) and DNA fragmentation is suggestive of PCD. In contrast, in another study of mortality, this time within various filament-forming freshwater cyanobacteria (Agustí et al., 2006), mortality was either absent or total within filaments, with nothing in between, suggesting “physiological integration” within filaments. Rychtecky et al. (2014) linked *Aphanizomenon* mortality to light availability and thereby highlighted the intuitive connection between resource availability and mortality. Individual *Microcystis* cells move within colonies perhaps driven by the need to meet individual physiological requirements (Mulling et al., 2014). Dead cells within colonies may be an adaptive feature of colony development, just as terminal differentiation within metazoans is recognized as a natural part of development. Differentiation within colonies/filaments for specific physiological purposes (e.g., akinetes and heterocytes) is well known and the capacity for this can be actively exploited by plants in a wide range of symbioses (Meeks and Elhai, 2002). In assessing free-living cyanobacteria, however, difficulties remain in the interpretation and categorization of cyanobacteria cell death. To date, several quite different methodologies have been applied across different taxa. Some of the methods used to determine “viability” (e.g., general hydrolytic enzyme activity assays such as FDA), by their nature, cannot deliver clear categorizations of cell physiological state. This is because of the huge variability in physiological state that will be encountered in any natural free-living microbial population. Variability in physiological state arises from the almost limitless possible range of individual cell, and colony, age and experience (Davey, 2011). The results from general enzyme assays, such as FDA, reflect this fact (e.g., Brookes et al., 2000) and in cyanobacteria, the existence of alternative physiological states to active proliferation (e.g., quiescent states, Sauer et al., 2001; Meireles et al., 2015) further confuse the interpretation of some viability assessment methods. Surveys of dead cyanobacteria cells in marine systems have also shown highly variable proportions of such cells (e.g., Alonso-Laita and Agustí, 2006; Elovaara et al., 2020; Vanharanta et al., 2020) which has been linked to mortality caused by photo-oxidative stress (Alonso-Laita and Agustí, 2006). The idea that irradiance, as a mediator of “photo-oxidation,” acts as an ultimate cause of cyanobacteria mortality is long established (e.g., see refs in Franklin et al., 2006) and irradiance would be amongst the first of the external factors recognized to cause cell death in cyanobacteria. Interestingly, potentially stressful irradiance conditions can also be linked with viral fitness given that some bacteriophages encode photosynthetic genes (Mann and Clokie, 2012). Using these genes, it is proposed that viral manipulation of host cell metabolism allows the continued production of viral progeny even under the conditions which would normally exceed cell oxidative stress theresholds (Clokie and Mann, 2006) and, presumably, lead to cell death.

### External Factors

The external factors that can cause cyanobacteria cell death are legion. The baroque ecological communities that develop during cyanobacterial bloom events (e.g., Woodhouse et al., 2016) contain a huge range of organisms that can negatively affect the blooming organism. The role of colony formation as an adaptation to reduce grazing pressure has been extensively studied (e.g., Haarke et al., 2016) and in the case of *Microcystis*, about 120 taxa, including viruses, bacteria, microfungi, other heterotrophic protists, other cyanobacteria, and several eukaryotic microalgal groups are known to negatively affect growth by infection and predation or by the production of allelopathic compounds (Van Wichelen et al., 2016) all of which could potentially result in mortality. Such external factors, which in the case of bacterial interactions may be mediated by signaling compounds (e.g., Weiss et al., 2019), are generally poorly known yet likely to be highly significant in driving the dynamics of cyanobacterial blooms as well as their rapid coevolution (“arms race”) with their microbial neighbors. Certain volatile organic compounds produced by cyanobacteria may have allelopathic qualities against other cyanobacteria, and have been linked with episodes of mass cell death and lysis (Arii et al., 2015) further emphasizing the complexity and importance of biotic interactions in driving population dynamics. It is clear that the observation of non-viable cells in complex natural populations may have very complex causes. Because of this, an accurate attribution of the cause(s) of cell death in natural cyanobacteria populations can only potentially be made with intensive temporal sampling (Kozik et al., 2019) combined with comprehensive assessments of abiotic conditions and accompanying biotic diversity.

## Regulated and Programmed Cell Death – Observations in Laboratory Populations

What can laboratory observations of cyanobacterial cell death teach us about cell death in the highly complex and variable conditions which natural populations both experience and create? Oxidative stress, caused by the presence of ROS can have severe effects on free-living bacteria (Ezraty et al., 2017) and research continues to focus on the links between oxidative stress and cell death. Cyanobacteria will experience ROS generated within their environment (e.g., by the action of UV on dissolved organic matter) as well as ROS generated within the cell via biochemistry. A particular vulnerability of cyanobacteria to the ROS H_2_0_2_ is now exploited in some bloom mitigation technologies. Few studies have partitioned environmental and endogenous sources, though Cory et al. (2016) found the biochemical source to be typically greater. Oxidant formation within the cell is not only linked to these two sources but also by disruptions to biochemical processes through the presence (or absence) of other growth-related compounds. In *Microcystis aeruginosa*, PCD has been diagnosed in the laboratory, or predicted, with reference to ROS stress (Ross et al., 2006; Ding et al., 2012) and the factors which would induce intracellular oxidant formation or exposure in nature have been speculated to lead to PCD in nature (Ross et al., 2006; Guo et al., 2012; Moon et al., 2012). This volume is focused on the possibility that such a “regulated” pathway – involving a collective and coordinated participation of a wide range of cellular processes – could, at times, control the massive lysis of natural cyanobacterial populations. Recent work on the role of ROS, and oxidative stress more generally, in causing a regulated form of cell death builds on the older idea of “photo-oxidative” cell death in cyanobacteria (e.g., Abeliovich and Shilo, 1972). The central role of internal imbalances leading to ROS generation, and then cell death, can be clearly demonstrated in the laboratory but assessing oxidative stress and the consequences for the cell *in situ* remain a critical challenge (Latifi et al., 2008). Recently, the variety of stimuli (in addition to oxidative stress) which can lead to *Microcystis* cell death in the laboratory – with the biochemical characteristics associated with PCD – were reviewed (Hu and Rzymski, 2019). These observations have led to some intriguing hypotheses on the possible role of PCD in *Microcystis* ecology (Hu and Rzymski, 2019). One of these hypotheses postulates that *Microcystis* PCD can be induced by an oxidative stress, such as UV, which leads to microcystin release. Since microcystins are known to stimulate colony formation (Gan et al., 2012) the cell death of some cells improves the overall fitness of the population by facilitating colony formation and thereby ensuring that overall cell (population) survival is more likely (Hu and Rzymski, 2019). This idea is supported by the increased expression of genes linked with colony-formation genes during microcystin exposures (Gan et al., 2012) and the notion that other cell constituents, released via cell lysis, could simply have a structural role in colony formation (with reference to observations on heterotrophic bacterial biofilms, see Hu and Rzymski, 2019). With respect to the rapid *Microcystis* population declines that can be observed in nature (Bozarth et al., 2010), however, as peaks in cyanophage activity can coincide with drastic reductions in *Microcystis* abundance, infection events appear to be common (Haarke et al., 2016), and highly strain-specific cyanophages can potentially adopt a lysogenic state (Mann and Clokie, 2012; Van Wichelen et al., 2016), the potential links between viral lysis and the biochemical markers associated with PCD (Bidle, 2015) may be the first point of scrutiny in understanding the causes of rapid *Microcystis* population declines.

## Gaps in Our Knowledge and Priorities for Study

As concern builds over the possible increase in the incidence of cyanobacterial blooms (Paerl and Huisman, 2009; Paerl and Paul, 2012) research is needed to understand if elevated abundances of freshwater cyanobacteria will translate into an increased incidence of cyanotoxin production and therefore risks to health (e.g., Paerl and Otten, 2013; c.f. Wilhelm and Boyer, 2011). Interestingly, a suggested link between microcystin (an important cyanotoxin) and the ability to withstand oxidative stress, and the proposed feedback between cell death and dissolved organic carbon (which has the potential to generate environmental ROS) are suggested (Paerl and Otten, 2013) as key factors in driving the selection for toxigenic over non-toxigenic strains (but see also Schuurmans et al., 2018). To improve understanding of such complex processes, with particular reference to the functioning of natural bloom situations, a future research focus is suggested in:

(1) High-resolution studies of cyanobacteria physiological state (mortality and oxidative stress, using consistent methods) in natural populations over the seasons, and over bloom cycles, accompanied by the molecular characterization of the viral and microbial communities which accompany the bloom, and the non-bloom, situation.

and,

(2) laboratory/culture studies (using fresh isolations and semi-natural continuous culture methods) investigating the performance of the methods available to characterize cell death, including biomarkers thought to be associated with internal pathways, and oxidative stress, in order to support the interpretation of 1).

This broadly echoes previous suggestions (Hu and Rzymski, 2019; Kozik et al., 2019) as to some priorities in this field. More fully understanding the role of mortality in natural cyanobacteria populations requires careful assessment of individual physiological state. Death is one possible, and fundamental, physiological state but its assessment is not straightforward in natural microbial populations. Any methods used must undergo careful validation (Davey and Guyot, 2020) with the “gold standard” or classical approach – i.e., the quantitative assessment of the proportion of cells that can proliferate after transfer to a benign artificial environment – basically unfeasible in environmental microbiology. Relatively simple protocols based on single-stain uptake (e.g., SYTOX-green, testing membrane permeability) combined with very rapid (or better, *in situ*) flow cytometry probably offer the most practical method of investigating the extent of cell death that may occur during rapid population fluctuations. This type of staining approach has found favor for heterotrophic bacteria (Roth et al., 1997; Shapiro, 2008) and is now relatively affordable. *In situ* flow cytometry has revolutionized our understanding of marine picocyanobacteria population dynamics (Hunter-Cevera et al., 2020) though this approach has not yet been attempted in freshwater microbiology. The colony or chain/filament forming habit of many freshwater cyanobacteria potentially complicates the use of cytometry although some cytometry systems have more potential than others in coping with colonies (Zhou et al., 2012). In terms of the biomarkers that have been applied to cyanobacteria to attempt a categorization of the cause of cell death (annexin, caspase activity, and TUNEL labeling; see Hu and Rzymski, 2019) difficulties are raised due to the findings of variable staining between taxa and the difficulties of interpretation due to the lack of satisfactory positive controls (Kozik et al., 2019). Whereas growth and chemical imbalances, and exogenous stress in the form of ROS exposure, can be shown to cause increases in such markers in laboratory culture work, evidence for the significance of such pathways in natural populations is poorly developed. We do know for sure though that a myriad of ecological interactions are likely to both interfere with cyanobacteria division in nature and potentially cause cell death (Van Wichelen et al., 2016) and the lesson from marine microbial ecology is that viral lysis is likely to be highly significant.

## Conclusion

Much of the work reviewed here concerns *Microcystis*, and the focus on *Microcystis* in the literature is unsurprising given its importance. It is inevitable that research focuses on the adaptations and life-histories of the “nuisance” forms which can dominate lake systems. Most of the <30 “nuisance” forms (Whitton and Potts, 2013) are colony or filament forming and represent some of the oldest forms of multicellularity on Earth. Colonies and chains/filaments make high resolution cytometric analysis more challenging but not impossible. Whereas it seems possible that inherent cyanobacteria cell death pathways, with no lysogenic, or other, viral component, can be activated in response to external stress, or are inherent in the fundamental nature of cell division, the evidence base for the significance of this in nature needs further development. Certainly, intriguing evolutionary arguments are possible and it’s hoped that the two studies which have so far attempted a simultaneous cell death and biomarker approach in natural *Microcystis* populations are joined by more as the use of field cytometry becomes more feasible and that such studies routinely characterize the accompanying viral and microbial communities. The dynamism and potency of freshwater cyanobacteria populations are attractive and form an ecological conundrum which clearly deserves significant research attention.

## Author Contributions

The author confirms being the sole contributor of this work and has approved it for publication.

## Conflict of Interest

The author declares that the research was conducted in the absence of any commercial or financial relationships that could be construed as a potential conflict of interest.
